# Correction: powerlaw: A Python Package for Analysis of Heavy-Tailed Distributions

**DOI:** 10.1371/journal.pone.0095816

**Published:** 2014-04-25

**Authors:** The PLOS ONE Staff

Figure 1 is incorrect. The authors have provided a corrected version here.

**Figure 1:**
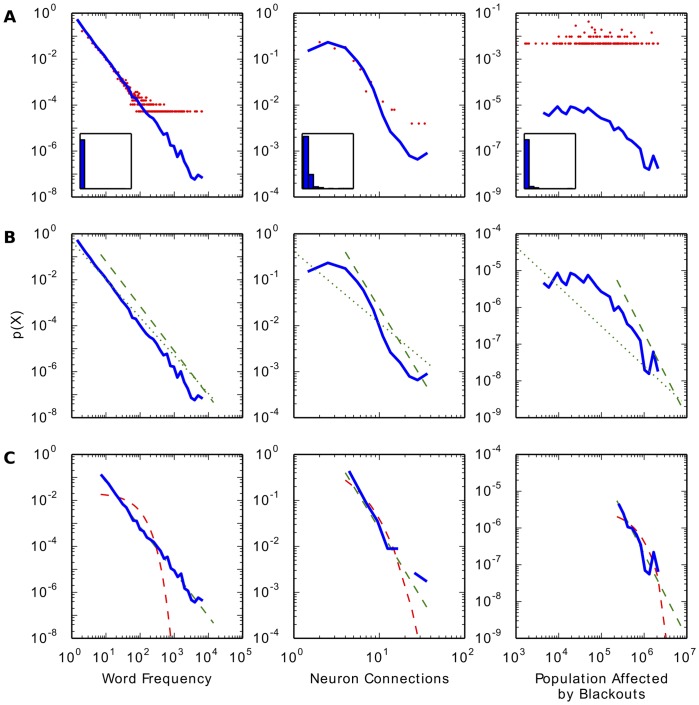
Basic steps of analysis for heavy-tailed distributions: visualizing, fitting, and comparing. Example data for power law fitting are a good fit (left column), medium fit (middle column) and poor fit (right column). Data and methods described in text. a) Visualizing data with probability density functions. A typical histogram on linear axes (insets) is not helpful for visualizing heavy-tailed distributions. On log-log axes, using logarithmically spaced bins is necessary to accurately represent data (blue line). Linearly spaced bins (red line) obscure the tail of the distribution (see text). b) Fitting to the tail of the distribution. The best fit power law may only cover a portion of the distribution's tail. Dotted green line: power law fit starting at  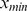  =  1. Dashed green line: power law fit starting from the optimal 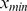 (see Basic Methods: Identifying the Scaling Range). c) Comparing the goodness of fit. Once the best fit to a power law is established, comparison to other possible distributions is necessary. Dashed green line: power law fit starting from the optimal 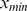. Dashed red line: exponential fit starting from the same 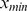.

Due to publisher error, the legend for Figure 2 appears incorrectly. The corrected version can be viewed below.

**Figure 2:**
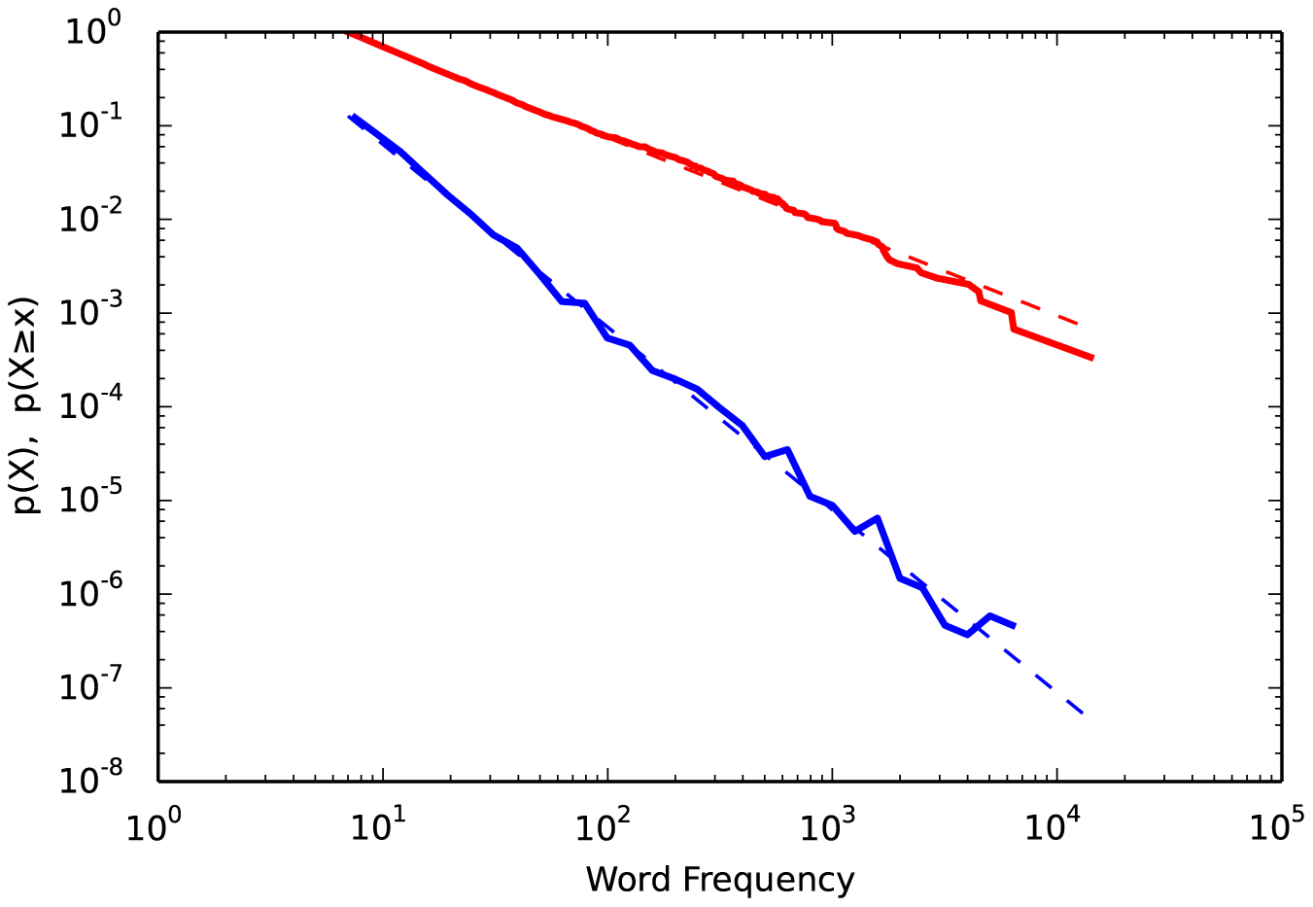
Probability density function (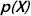, blue) and complemenatary cumulative distribution function (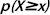, red) of word frequencies from "Moby Dick".
