# Supplementary figures and images for: powerlaw: A Python Package for Analysis of Heavy-Tailed Distributions
Source: PLoS One. 2014 Jan 29;9(1):e85777. doi: 10.1371/journal.pone.0085777 (PMC3906378; doi:10.1371/journal.pone.0085777)

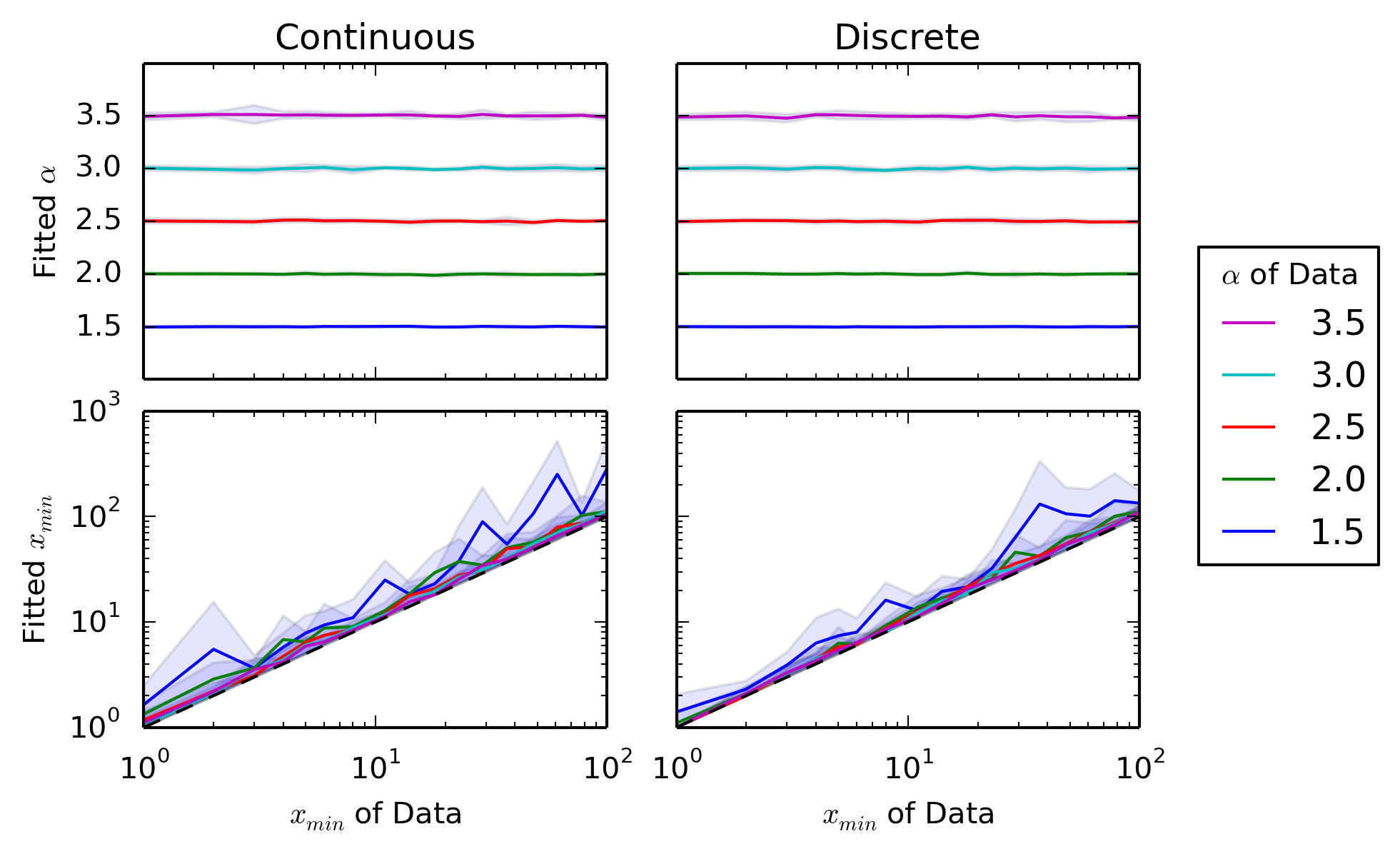

Supplement: Figure S1 — Validation of fitting accuracy on simulated data with different values of and . Each fit is the average of 10 simulated datasets of 10,000 data points each. Shading is the standard deviation of the 10 simulations. Note that on these simulated data there exist no data smaller than the true from which to sample, so any statistical fluctuation in the estimation of must return a value larger than the true value. The black dashed line on the bottom panels is the boundary where the fitted is equal to the actual , below which fits cannot be made. For datasets in which there are noisy data below the of the power law, these methods recover the even more accurately, as shown in [5]. (TIFF) [file pone.0085777.s004.tiff]
